# Case Report: Acute arterial occlusion of the right lower extremity due to left atrial invasion from pulmonary metastases of thyroid cancer

**DOI:** 10.3389/fcvm.2023.1221180

**Published:** 2023-11-30

**Authors:** Kentaro Akabane, Tetsuo Watanabe, Yuta Tajima, Shuji Toyama, Yoshihisa Tamate, Tetsuro Uchida

**Affiliations:** ^1^ Division of Cardiovascular Surgery, Sendai City Hospital, Miyagi, Japan; ^2^ Second Department of Surgery, Faculty of Medicine, Yamagata University, Yamagata, Japan

**Keywords:** acute arterial occlusion, lower extremity, thyroid cancer, multiple pulmonary metastases, left atrial invasion

## Abstract

Cardiac metastases of thyroid cancer are rare. The most common metastatic route is through lymphatic or hematogenous spread to the right side of the heart. Direct invasion of metastases from other adjacent organs to the left side of the heart is even rarer. In many cases, the disease progresses asymptomatically, and symptoms appear only when it is already fatal. A 68-year-old woman underwent total thyroidectomy and right-side lymph node dissection for papillary thyroid cancer and multiple lung metastases 7 years previously. The patient was referred to our hospital due to sudden pain in the right lower extremity and motor disturbance. Computed tomography revealed acute arterial occlusion of the right lower extremity due to tumor dispersal from a left atrial invasion caused by multiple pulmonary metastases of thyroid cancer, and only emergency thrombectomy was performed. Although blood flow was restored, the patient died of respiratory failure 2 months after the procedure. Radical resection is considered difficult in cases of direct invasion of metastases from other adjacent organs because multiple metastases have often already occurred. Therefore, in the terminal stage, it might be too invasive to resect a tumor only to prevent embolism recurrence. The treatment strategy should depend on the patient's prognosis and choice.

## 1. Introduction

Cardiac metastases of thyroid cancer are rare (1, 2). The most common metastatic route is through lymphatic or hematogenous spread to the right side of the heart (3). Direct invasion of metastases from other adjacent organs to the left side of the heart is even rarer (4). In many cases, the disease progresses asymptomatically and is already at the terminal stage when symptoms appear (5). Therefore, invasive interventions might sometimes be considered over-treatment, and it is thus necessary to consider the treatment strategy according to individual conditions. We report a case of acute arterial occlusion of the right lower extremity due to tumor dispersal from left atrial invasion by multiple pulmonary metastases of thyroid cancer for which a thrombectomy was performed. We also review the treatment strategy.

## 2. Case description

A 68-year-old woman underwent total thyroidectomy and right-side lymph node dissection for papillary thyroid cancer 7 years ago. The tumor, measuring approximately 2.5 cm, was located in the right lobe isthmus and had partially invaded the trachea. Additionally, two intralobular metastases, measuring approximately 8 mm, were identified in the left lobe. Because multiple lung metastases were detected preoperatively, the patient was treated with oral radioactive iodine postoperatively. Subsequent scintigraphy showed no significant accumulation in the lung lesions; hence, no additional treatment with oral radioactive iodine was administered. During follow-up, it was observed that the lung metastases were slowly spreading, leading to the initiation of oral multikinase inhibitors for the patient 2 years ago.

The patient was referred to our hospital 30 min after the onset of abrupt right lower extremity pain and motor disturbance. On arrival, the patient exhibited severe hypertension, probably due to pain, while remaining clinically stable (blood pressure, 185/117 mmHg; heart rate, 98 bpm; respiratory rate, 20 /min; SpO_2_, 94%). However, the right lower extremity was cold and cyanotic, and only the common femoral artery (CFA) pulsation was faintly palpable. The electrocardiography showed normal sinus rhythm, while the bedside echocardiography revealed a mass in the left atrium. Although the images were unclear, the diameter of the mass was approximately 3 cm. The ejection fraction was approximately 60%, and no significant valvular findings were observed.

**Figure 1:**
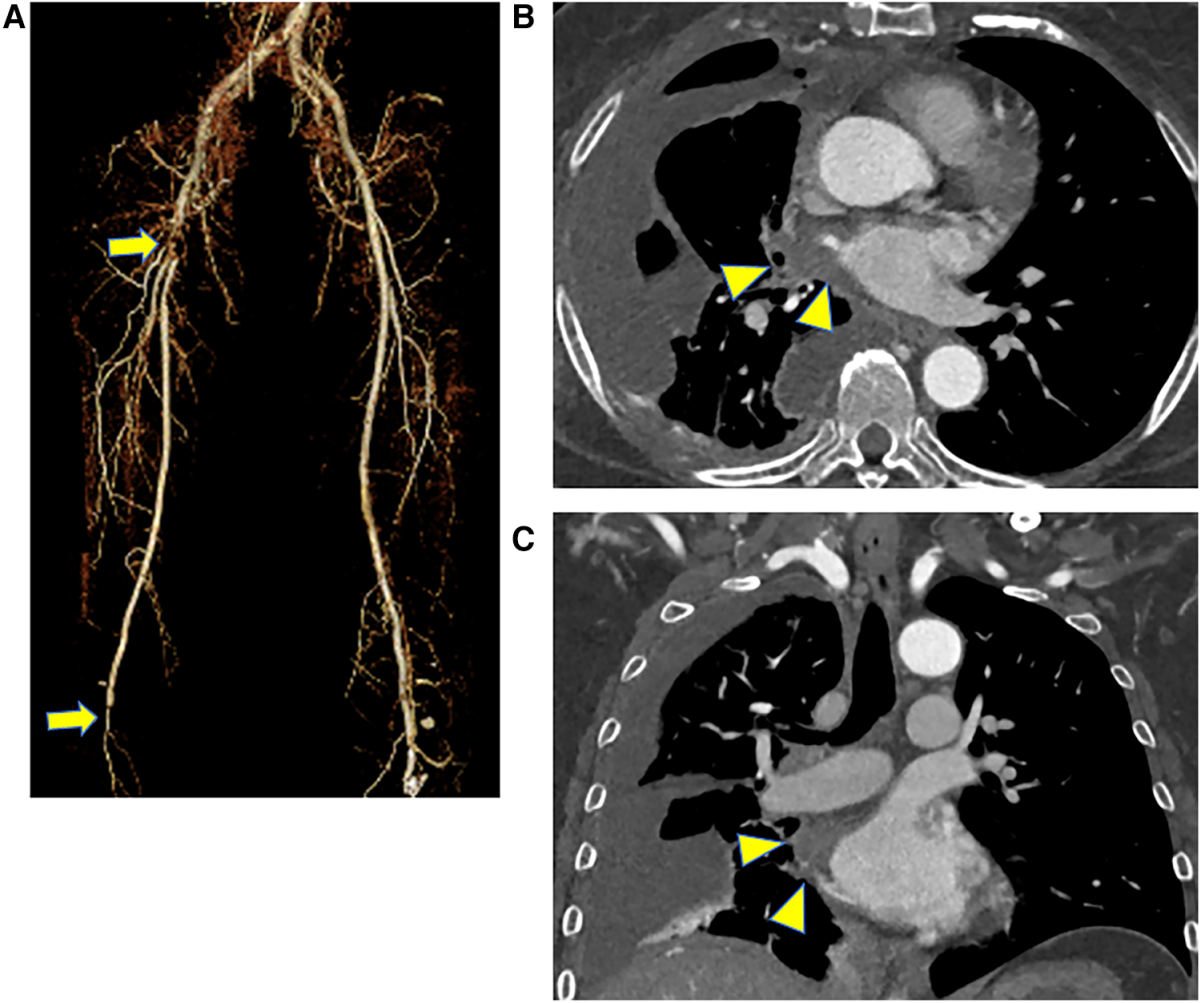
Preoperative CT scans, (**A**) three-dimensional CT of the lower extremity, and (**B,C**) CT of the chest level. CT showed contrast deficits from the right CFA to the proximal SFA and DFA and at the popliteal artery behind the knee (arrow). There was also a contrast defect from the right pulmonary vein into the left atrium, which might be a thrombus with a direct invasion of pulmonary metastases (arrowhead). CT, computed tomography; CFA, common femoral artery; SFA, superior femoral artery; DFA, deep femoral artery.

Computed tomography showed contrast deficits from the right CFA to the proximal superior femoral artery (SFA) and deep femoral artery (DFA) and at the popliteal artery behind the knee (Figure 1A). The pulmonary metastases were prominently enlarged compared to the condition 1 month earlier. Moreover, a contrast defect was observed from the right pulmonary vein into the left atrium, suspected to be a thrombus with direct invasion of the pulmonary metastases (Figures 1B,C).

**Figure 2:**
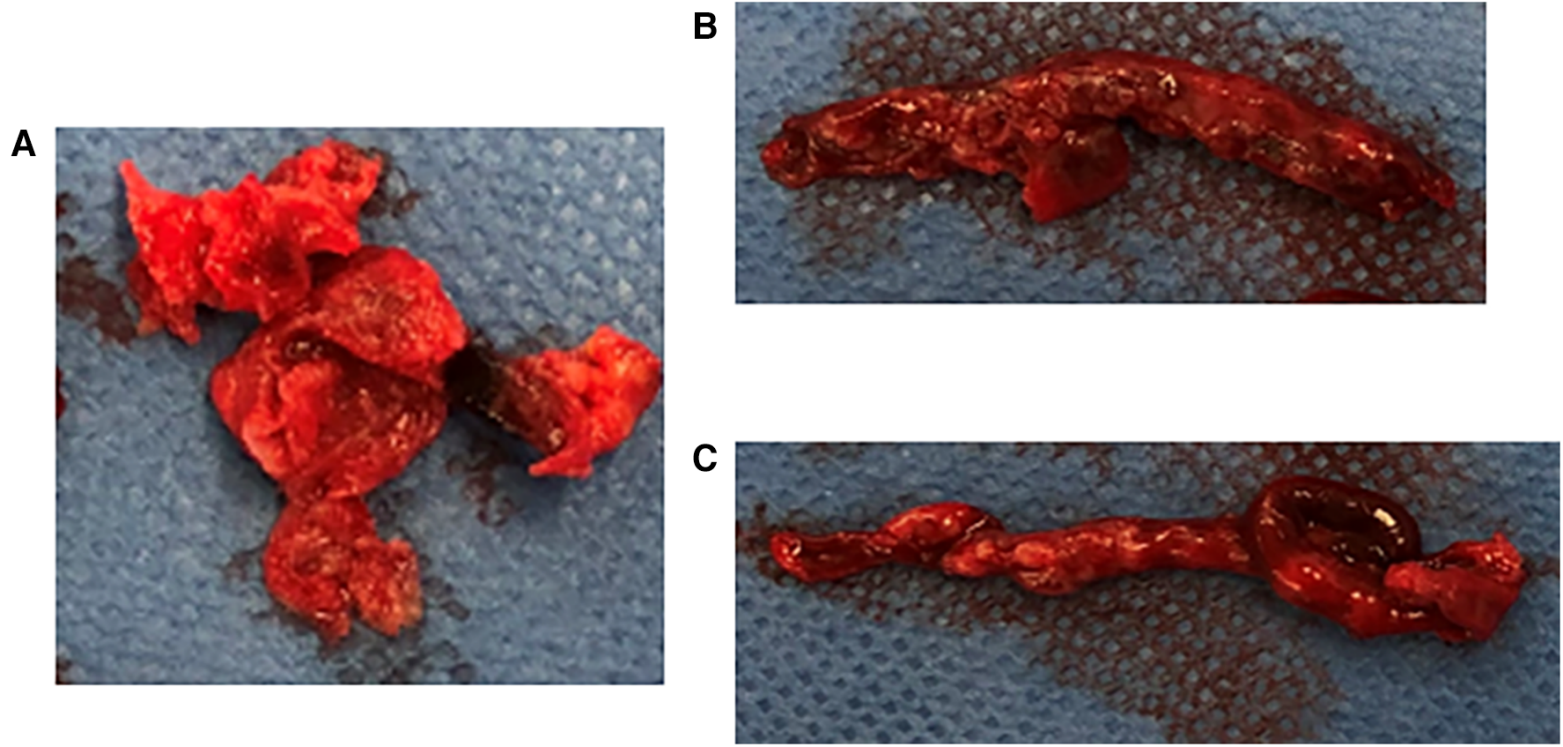
Embolus removed intraoperatively, (**A**) yellow solid mass removed from the CFA, (**B,C**) black thrombus removed from the SFA and DFA. The CFA was found to be filled with a yellow solid mass during the incision. The black thrombi were removed from the SFA and DFA. CFA, common femoral artery; SFA, superior femoral artery; DFA, deep femoral artery.

**Figure 3:**
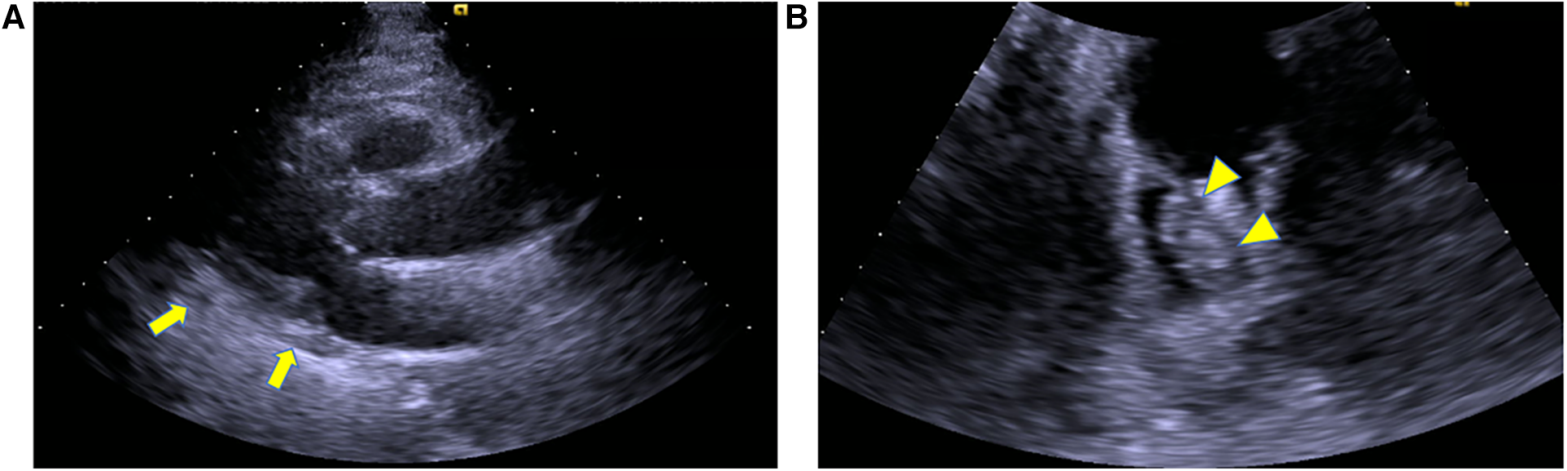
Postoperative echocardiography, (**A**) left ventricular long axis view, (**B**) 4 chamber view.echocardiography showed no pericardial effusion (arrow). It showed a floating mass extending from the left atrial orifice of the right pulmonary vein toward the atrial septum, which was considered a potential embolic source (arrowhead).

Emergency surgery was planned with a diagnosis of acute ischemia of the right lower extremity (category IIb) due to suspected tumor dispersal from the left atrium. After administration of local anesthesia, the CFA, SFA, and DFA were exposed through an inguinal skin incision. The CFA was found to be filled with a yellow solid mass during the incision (Figure 2A), and black thrombi were removed from the SFA and DFA (Figures 2B,C). Blood flow resumed 5 h after onset, and apixaban was started the day after surgery. The postoperative course was uneventful. Although postoperative echocardiography showed no local wall motion abnormalities and pericardial effusion, it showed a floating mass ≥3 cm extending from the left atrial orifice of the right pulmonary vein toward the atrial septum. Considering the size and location, it was the same mass observed preoperatively and thought to be a potential embolic source (Figures 3A,B). Computed tomography revealed good blood flow to the periphery, and the patient was discharged 17 days postoperatively.

**Figure 4:**
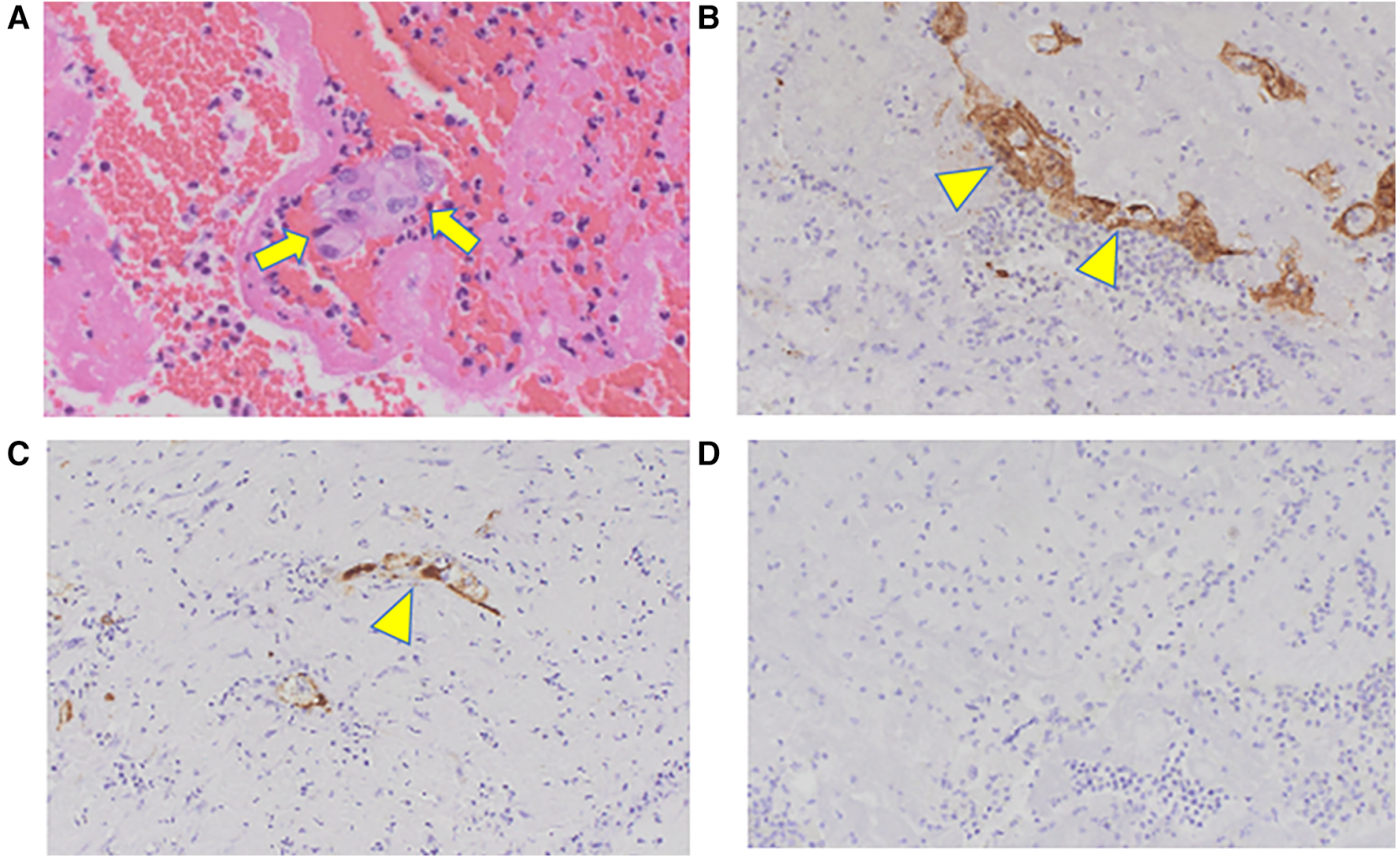
Pathological findings: (**A**) HE staining, (**B**) CK AE1/AE3, (**C**) CAM5.2, (**D**) TTF-1. The yellow embolus removed intraoperatively shows atypical cells on HE staining (arrow). The cells are typical of epithelial carcinoma cells because CK AE 1/AE 3 and CAM 5.2 are stained positive (arrowhead). TTF-1 is stained negative, indicating undifferentiated metastasized thyroid cancer. HE, Hematoxylin and eosin; CK, cytokeratin; TTF-1, thyroid transcription factor-1.

The yellow embolus removed intraoperatively had atypical cells on hematoxylin and eosin staining (Figure 4A). Additional immunostaining was performed; the cells were determined to be epithelial carcinoma cells because cytokeratin AE 1/AE 3 (Figure 4B) and CAM 5.2 (Figure 4C), which are stained positive in most epithelial cells, were stained positive. Thyroid transcription factor-1 (TTF-1), normally stained positive in thyroid cancer, was stained negative (Figure 4D); however, TTF-1 is stained negative in undifferentiated carcinoma. Considering these results and the rapid clinical course, the original papillary thyroid carcinoma might have transformed into undifferentiated carcinoma, which quickly invaded the left atrium from the lung metastases, and an embolism had developed. Although myocardial enzyme levels were not elevated and obvious signs of heart failure were absent during hospitalization, shortness of breath due to the progression of pulmonary metastases gradually increased shortly after that. The patient died of respiratory failure 2 months after surgery.

## 3. Discussion

Cardiac metastases of malignant tumors are rare, and the most common metastases of primary tumors are carcinomas of the lung and breast (1, 2). Cardiac metastases of thyroid cancer are even less common, with only 54 cases reported in the 130 years between 1881 and 2010, most of which were diagnosed during postmortem autopsy (6). This may be attributed to the fact that most cases progress asymptomatically, and patients are at the terminal stage when symptoms appear (5). Therefore, cardiac metastases of thyroid cancer are difficult to diagnose while the patient is alive because cancerous tissues are rarely obtainable from any part of the body after symptoms manifest. The patient in the present case also had no obvious signs of cardiac failure. Hence, if acute arterial occlusion had not developed, the rapid spread of pulmonary metastases and cardiac invasion would not have been diagnosed before the patient's death. The most common routes of metastasis from the thyroid gland to the heart involve lymphatic or hematogenous spread to the right side of the heart via the vena cava (3). In contrast, metastases to the left side of the heart by direct invasion from metastases in other organs adjacent to the heart have rarely been reported (7–12).

To the best of our knowledge, this is the first reported case of acute lower extremity ischemia caused by the dispersal of a tumor invading the left atrium. Some studies have reported successful complete resection of the metastatic sites in cases of lymphatic or hematogenous spread to the right side of the heart via the vena cava (13, 14). Tumor resection should be performed if radical resection is possible and the long-term prognosis is favorable. However, in cases of direct invasion to the left side of the heart due to metastases from other organs, radical resection is regarded as challenging because multiple metastases have often already occurred (4). Considering the invasiveness of surgery and the possibility of metastatic spread due to the cardiopulmonary bypass (15), it is unclear whether tumor resection to prevent embolism is appropriate for terminal-stage prognosis, as it may sometimes be over-invasive. In our case, resection of the tumor was deemed too invasive, and only revascularization was performed. However, there was a concern that embolization might recur. Since no definitive treatment policy exists for such cases, it is necessary to consider a treatment strategy according to the patient's prognosis and choice.

## Data Availability

The original contributions presented in the study are included in the article/Supplementary Material, further inquiries can be directed to the corresponding author.

## References

[1] Abraham KP Reddy V Gattuso P. Neoplasms metastatic to the heart: review of 3314 consecutive autopsies. Am J Cardiovasc Pathol. (1990) 3:195–8. 2095826

[2] Klatt EC Heitz DR. Cardiac metastases. Cancer. (1990) 65:1456–9. 10.1002/1097-0142(19900315)65:6<1456::AID-CNCR2820650634>3.0.CO;2-5 2306690

[3] Niederle B Hausmaninger C Kretschmer G Polterauer P Neuhold N Mirza DF Intraatrial extension of thyroid cancer: technique and results of a radical surgical approach. Surgery. (1990) 108:951–6. 2247840

[4] Thompson NW Brown J Orringer M Sisson J Nishiyama R. Follicular carcinoma of the thyroid with massive angioinvasion: extension of tumor thrombus to the heart. Surgery. (1978) 83:451–7. 635781

[5] Giuffrida D Gharib H. Cardiac metastasis from primary anaplastic thyroid carcinoma: report of three cases and a review of the literature. Endocr Relat Cancer. (2001) 8:71–3. 10.1677/erc.0.0080071 11350728

[6] Catford SR Lee KT Pace MD Marasco SF Longano A Topliss DJ. Cardiac metastasis from thyroid carcinoma. Thyroid. (2011) 21:855–66. 10.1089/thy.2010.0273 21751883

[7] Rühli FJ Hilfiker PR. Metastasis of thyroid cancer to the heart. Am J Roentgenol. (2001) 177(2):474. 10.2214/ajr.177.2.1770474 11461895

[8] Shai SE Hsieh SR Song YM Shen GH. Resection of follicular thyroid cancer metastasized to the left lower lobe of the lung extending into the left atrium as a huge intracardiac tumor. Thyroid. (2005) 15:1417–8. 10.1089/thy.2005.15.1417 16405423

[9] Bertoldi EG Severo MD Scheffel RS Foppa M de Azevedo MJ Maia AL. Left atrial metastases of poorly differentiated thyroid carcinoma diagnosed by echocardiography and magnetic resonance imaging—case report and review of literature. Echocardiography. (2012) 29:30–3. 10.1111/j.1540-8175.2011.01549.x 22044639

[10] Giovanella L Treglia G Ceriani L Weidner S Perriard U Bongiovanni M. Left atrial metastasis of Hürthle-cell thyroid carcinoma mimicking myxoma. J Nucl Cardiol. (2014) 2:406–7. 10.1007/s12350-013-9826-8 24263637

[11] Sood A Parihar AS Sood A Kumar R Prakash G Singhal M Unusual presentation of Hurthle cell carcinoma with TENIS syndrome as left atrial thrombus on 18F-FDG PET/CT. Clin Nucl Med. (2018) 43:352–4. 10.1097/RLU.0000000000002224 30080688

[12] Cassar MP Strirrup J. Left atrial tumor thrombus in metastatic thyroid cancer. J Cardiovasc Comput Tomogr. (2020) 14:155–6. 10.1016/j.jcct.2019.12.005 31836413

[13] Kaul S Tulchinsky M Campbell DB Crist HS Manni A. Isolated cardiac metastasis from papillary thyroid cancer: prolonged survival with late diagnosis related to inadequate positron emission tomography preparation. Thyroid. (2012) 22:443–4. 10.1089/thy.2011.0295 22376168

[14] Tricard J Chermat A Abdelkafi E Piccardo A. Giant intracardiac medullary thyroid cancer metastasis. J Card Surg. (2022) 37:5455–6. 10.1111/jocs.17162 36423260

[15] Hasegawa S Otake Y Bando T Cho H Inui K Wada H. Pulmonary dissemination of tumor cells after extended resection of thyroid carcinoma with cardiopulmonary bypass. J Thorac Cardiovasc Surg. (2002) 124:635–6. 10.1067/mtc.2002.125060 12202885

